# A warmer environment can reduce sociability in an ectotherm

**DOI:** 10.1111/gcb.16451

**Published:** 2022-10-19

**Authors:** Natalie Pilakouta, Patrick J. O'Donnell, Amélie Crespel, Marie Levet, Marion Claireaux, Joseph L. Humble, Bjarni K. Kristjánsson, Skúli Skúlason, Jan Lindström, Neil B. Metcalfe, Shaun S. Killen, Kevin J. Parsons

**Affiliations:** ^1^ School of Biodiversity, One Health and Veterinary Medicine University of Glasgow Glasgow UK; ^2^ School of Biological Sciences University of Aberdeen Aberdeen UK; ^3^ Department of Biology University of Turku Turku Finland; ^4^ Department of Biological Sciences University of Montreal Montreal Canada; ^5^ Norwegian Institute of Marine Research Bergen Norway; ^6^ Department of Aquaculture and Fish Biology Hólar University Sauðárkrókur Iceland; ^7^ Icelandic Museum of Natural History Reykjavík Iceland

**Keywords:** behavioural reaction norm, climate change, *Gasterosteus aculeatus*, genotype‐by‐environment interaction, phenotypic plasticity, shoaling, sociality, thermal effects, threespine stickleback

## Abstract

The costs and benefits of being social vary with environmental conditions, so individuals must weigh the balance between these trade‐offs in response to changes in the environment. Temperature is a salient environmental factor that may play a key role in altering the costs and benefits of sociality through its effects on food availability, predator abundance, and other ecological parameters. In ectotherms, changes in temperature also have direct effects on physiological traits linked to social behaviour, such as metabolic rate and locomotor performance. In light of climate change, it is therefore important to understand the potential effects of temperature on sociality. Here, we took the advantage of a ‘natural experiment’ of threespine sticklebacks from contrasting thermal environments in Iceland: geothermally warmed water bodies (warm habitats) and adjacent ambient‐temperature water bodies (cold habitats) that were either linked (sympatric) or physically distinct (allopatric). We first measured the sociability of wild‐caught adult fish from warm and cold habitats after acclimation to a low and a high temperature. At both acclimation temperatures, fish from the allopatric warm habitat were less social than those from the allopatric cold habitat, whereas fish from sympatric warm and cold habitats showed no differences in sociability. To determine whether differences in sociability between thermal habitats in the allopatric population were heritable, we used a common garden breeding design where individuals from the warm and the cold habitat were reared at a low or high temperature for two generations. We found that sociability was indeed heritable but also influenced by rearing temperature, suggesting that thermal conditions during early life can play an important role in influencing social behaviour in adulthood. By providing the first evidence for a causal effect of rearing temperature on social behaviour, our study provides novel insights into how a warming world may influence sociality in animal populations.

## Graphical abstract

We tested the effect of temperature on social behaviour using threespine sticklebacks from geothermally warmed lakes and adjacent ambient‐temperature lakes in Iceland. Using a common garden breeding design, we show that sociability is both heritable and influenced by rearing temperature, suggesting that thermal conditions during early life can play an important role in influencing sociability in adulthood. Our study provides the first evidence for a causal effect of temperature on social behaviour, offering novel insights into how a warming world may influence sociality in animal populations.

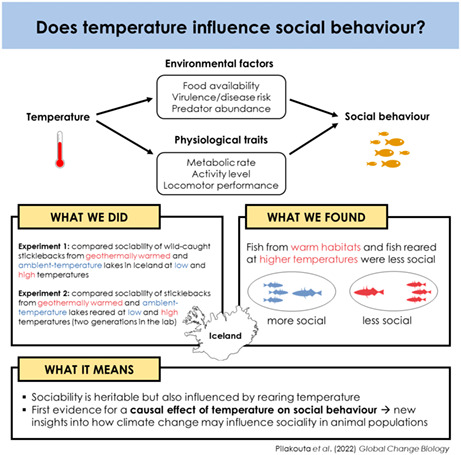

## 1 INTRODUCTION

The formation of animal groups, known as sociality, occurs in a wide range of taxa (Krause & Ruxton, 2002), but there is remarkable variation in the extent of social behaviour both among and within species (Cote et al., 2012; Webster & Laland, 2015). This variation can be partly explained by the fact that the costs and benefits of sociality are environment‐dependent (Chapman et al., 1995; Schradin et al., 2010). For example, animals living in groups benefit from improved predator detection and avoidance (Krause & Ruxton, 2002), so sociality may be favoured in environments with high predation pressure. On the other hand, sociality is associated with a higher rate of disease transmission among group members (Altizer et al., 2003). Being social may thus be more costly in environments with a high abundance of parasites or risk of infectious diseases. Group members may also benefit from a higher rate of food patch discovery (Ekman & Hake, 1988; Ruxton et al., 1995), but at the same time they might experience increased competition for discovered food items, resulting in intragroup aggression (Webster & Hart, 2006).

Through its effects on these and other ecological factors, temperature may play a key role in driving variation in social behaviour by altering the balance between the costs and benefits of sociality. For example, individuals may be less social in warmer environments due to a higher rate of disease transmission and increased virulence (Altizer et al., 2013; Harvell et al., 2002), which would make social interactions more costly. Warmer environments may also differ in food availability (Dillon et al., 2010; O'Connor et al., 2009) or predator abundance (Barbeau & Scheibling, 1994; Grigaltchik et al., 2016).

In addition, changes in ambient temperature can have direct effects on physiological traits in ectotherms, which can in turn influence their sociability (Cooper et al., 2018; Killen et al., 2016), defined as the propensity to associate with conspecifics. For example, a temperature‐induced increase in metabolic rate, and thus a higher energetic demand, might lead to more competition for food items and lower sociability. This prediction is supported by earlier work showing that hunger level is negatively correlated with social behaviour (Hansen et al., 2016; Krause et al., 2011). An increase in temperature is also associated with enhanced locomotor performance in fishes (Domenici et al., 2019), which may improve predator escape responses and thus reduce one of the main benefits of belonging to a group (i.e., reduced predation risk).

Understanding the potential relationship between temperature and social behaviour is especially important in light of global climate change (Moss & While, 2021). Even though rising temperatures could have profound consequences for the maintenance and diversification of social organization, these effects remain unknown and underappreciated (Moss & While, 2021). So far, the few studies on this topic have focused on phylogenetic approaches to look at the relationship between past climatic conditions and the occurrence of sociality across closely related species (e.g., Groom & Rehan, 2018; Jezovit et al., 2020). However, we still lack experimental studies testing whether similar processes could unfold within a single species or population on ecological timescales (Moss & While, 2021). In particular, common garden or reciprocal transplant experiments could help us untangle the role of plasticity and local adaptation in mediating changes in sociality in response to temperature variation (Fisher et al., 2021).

To address this knowledge gap, we took advantage of a ‘natural experiment’ of freshwater fish populations inhabiting contrasting thermal environments in Iceland: threespine sticklebacks (*Gasterosteus aculeatus*) in geothermally warmed water (warm habitats) and adjacent ambient‐temperature water (cold habitats). Sticklebacks display a shoaling behaviour like many other fish species, and there are consistent individual differences in their propensity to shoal (Jolles et al., 2017). In our first experiment, we measured the sociability of wild‐caught individuals from a pair of sympatric and a pair of allopatric warm and cold habitats that were acclimated to both low (10°C) and high (18°C) temperatures. We used a repeated measures design to obtain behavioural reaction norms, which allowed us to investigate (i) whether individuals from the warm habitats were more or less social than those from the cold habitats, (ii) whether sociability was phenotypically plastic in response to water temperature, and (iii) whether the degree of temperature‐induced plasticity in sociability differed between individuals from the warm and cold habitats (i.e., genotype‐by‐environment interaction). For our second experiment, we used a common garden breeding design, where individuals from the allopatric warm and cold habitats were reared from hatching at a low (12°C) and high (18°C) temperature for two generations. This approach allowed us to determine (i) whether rearing temperature influenced sociability and (ii) whether sociability, and the effects of rearing temperature on sociability, were heritable. By answering these questions, this study advances our understanding of how a warmer environment due to climate change may affect sociality, which can in turn have profound implications for group resilience and population persistence (Maldonado‐Chaparro & Chaverri, 2021).

## 2 METHODS

### 2.1 Study populations

**FIGURE 1:**
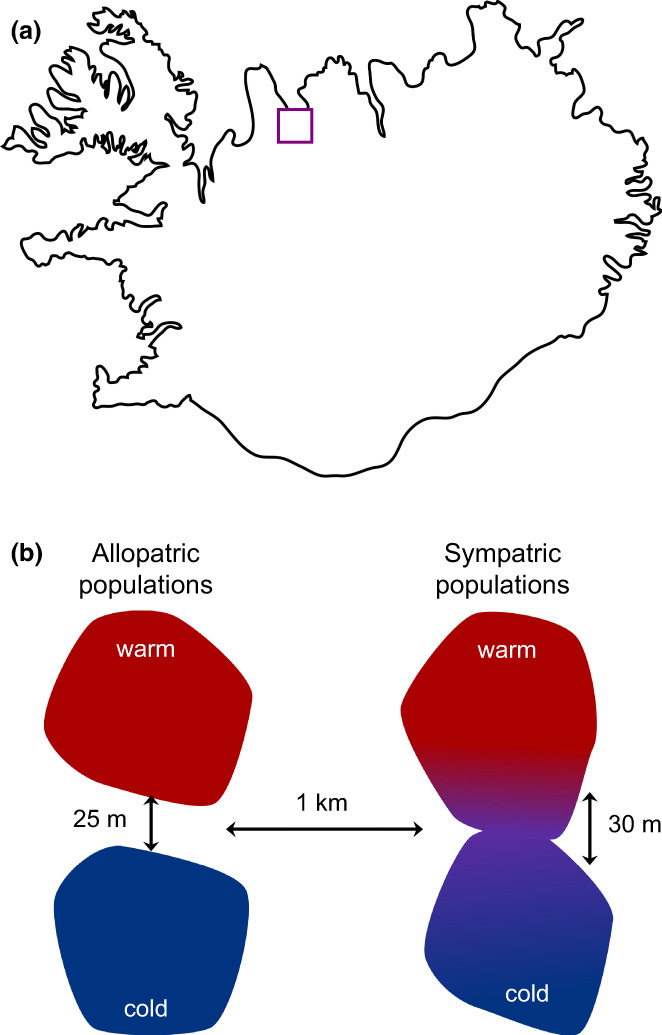
(a) Geographical location of stickleback populations used in this study as indicated by the purple square and (b) visualization of allopatric and sympatric thermal habitats. Distances indicate how far apart the two population pairs are from each other, as well as how far apart the warm‐habitat (red) and cold‐habitat (blue) sample sites are within each population pair (not drawn to scale).

In March 2017, we used unbaited minnow traps to collect freshwater threespine sticklebacks from two warm‐cold population pairs in Iceland (Figure 1a; *n* = 100 per sampling site, total *n* = 400). One of these population pairs was allopatric, meaning that the warm and cold habitats were in adjacent but separate water bodies with no potential for gene flow (Table S1; Figure 1b). The other population pair was sympatric, meaning that the warm and cold habitats were in the same water body with no physical barriers between them (Table S1; Figure 1b). Our preliminary population genomic analyses indicate extensive gene flow between thermal habitats in the sympatric population pair (Costa et al., in review). Previous work has shown morphological divergence between sympatric populations (Pilakouta, Humble, et al., 2022) but limited divergence in metabolic rate (Pilakouta, et al., 2020).

The cold habitats have existed for thousands of years since the last glacial period (Einarsson et al., 2004). The warm habitats have originated relatively recently (i.e., 50–70 years ago), fed by excess hot water runoff from nearby residences using geothermal heating. Since the generation time for threespine sticklebacks is about 1 year, the age of the warm habitats corresponds to the maximum number of generations each population pair may have been separated.

### 2.2 Transport of wild‐caught sticklebacks

Before transport to University of Glasgow, we fasted sticklebacks for 48 h to minimize the build‐up of ammonia in the transport water. On the day of shipping, we placed approximately 100 sticklebacks in each 100‐L polyethylene bag containing 25 L of water. Air was removed from the bags and replaced with pure oxygen. Bags were sealed and placed inside insulated Styrofoam shipping boxes to minimize temperature fluctuations during transport. The fish were in transit for approximately 72 h before arriving in Glasgow. No mortality was observed during transport.

### 2.3 Animal husbandry for wild‐caught sticklebacks

Once these fish arrived at University of Glasgow, they were kept at densities of approximately 15 individuals per 10‐L tank in a common recirculation system. Half of the fish from each population were placed at 10°C and the other half at 18°C. The low temperature (10°C) corresponds to a temperature that is intermediate between the annual extremes experienced by cold populations throughout the year, whereas the high temperature (18°C) corresponds to an intermediate temperature experienced by warm populations (Table S1). All fish were acclimated to these temperatures for at least 1 month prior to behavioural observations. During the acclimation period, fish were anaesthetized using benzocaine and marked with visible implant elastomer tags (Northwest Marine Technology Inc.) to allow individual identification. Fish were fed *ad libitum* twice a day with a mixture of frozen bloodworms, *Mysis* shrimp and *Daphnia*. They were kept at a 12 h light:12 h dark photoperiod throughout the experiment. All tanks contained plastic plants as shelter.

### 2.4 Experimental protocol for measuring sociability of wild‐caught sticklebacks

**FIGURE 2:**
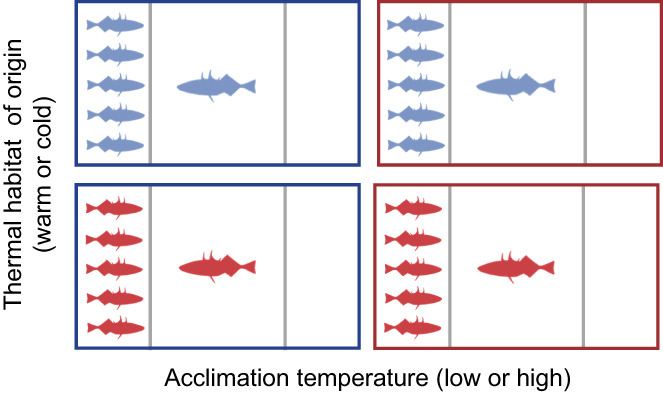
Experimental set‐up (not drawn to scale) for measuring sociability in wild‐caught sticklebacks from cold habitats (blue fish) and warm habitats (red fish). A focal fish was placed in the middle compartment, while a group of stimulus fish from the same thermal habitat was placed behind one of the two transparent dividers (grey lines). The side of the stimulus fish was determined randomly for each behavioural trial. Each individual was tested twice at a low temperature (blue tank) and twice at a high temperature (red tank). Fish were acclimated to the test temperature for at least 1 month prior to testing.

We measured the sociability of each individual using a binary choice test, where the focal individual could choose to spend more time near a compartment containing a stimulus shoal of conspecifics or another compartment that was left empty (Killen et al., 2016). Sociability was defined as the mean distance of the focal individual from the stimulus shoal over a 30 min trial. We used a rectangular tank (60 cm L × 31 cm W × 31 cm H) filled with water to a depth of 12 cm (Figure 2). This experimental tank was divided into three compartments using transparent acrylic partitions: a larger central arena (32 cm L × 31 cm W) and two smaller compartments on each side (14 cm L × 31 cm W). These partitions allowed visual and chemical cues but no physical interaction between the focal and stimulus fish. All sociability trials were recorded using a webcam (Logitech C920 HD Pro) mounted 60 cm over the central arena of the tank.

At the beginning of each trial, we placed a group of five stimulus fish into one of the side compartments, determined randomly using a coin toss. The other side compartment was left empty. After allowing the stimulus fish to acclimate for 5 min, we placed the focal fish into a glass cylinder in the central arena of the experimental tank. The focal and stimulus fish were unfamiliar with each other but were always from the same thermal habitat (Figure 2). Fish used as stimulus fish were never used as focal fish in other trials. We allowed the focal fish to acclimate for 5 min, at which point we lifted the glass cylinder and started the video recording. We recorded the movements of the focal individual for 30 min. After removing the focal and stimulus fish, we emptied the experimental tank and replaced the water before starting the next sociability trial.

Each individual was tested twice at their acclimation temperature (10°C or 18°C), with 7–10 days between trials. After completing both trials at one of these temperatures, fish were acclimated to the other temperature for at least 1 month, before being tested twice at this new temperature. Due to some mortality over the course of the study, a small proportion of these fish were only tested at either 10°C or 18°C (Table S1). Our sample sizes for this first experiment were between 28 and 32 individuals per collection site per acclimation temperature (total *n* = 250; Table S1).

### 2.5 Common garden breeding design

**FIGURE 3:**
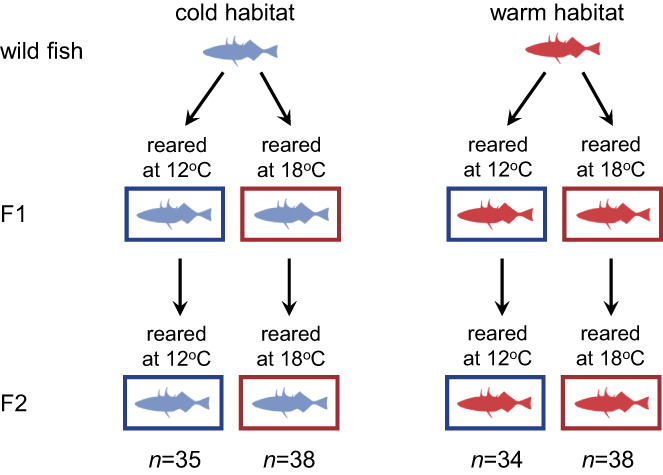
Breeding design for common garden experiment using the allopatric pair of stickleback populations. Using in vitro fertilization, we bred wild‐caught adults from a cold (blue) and a warm (red) habitat and reared their offspring (F1) at a low and a high temperature (12°C and 18°C, respectively). When these F1 individuals reached adulthood, we again performed in vitro fertilization and reared the F2 offspring at the same temperature as their F1 parents. When these F2 individuals reached adulthood, we measured the sociability of each individual twice at their rearing temperature. Rearing fish at a common temperature for two generations allowed us to minimize parental and other epigenetic effects.

We carried out a common garden experiment to test whether the differences we observed in sociability between the allopatric cold and warm populations were heritable or due to thermal conditions experienced in early life. For this experiment, we bred wild‐caught sticklebacks from the allopatric cold and warm habitats, which had been kept at a low (12°C) or high temperature (18°C), respectively (Figure 3). After performing in vitro fertilization in petri dishes, we placed fertilized embryos in mesh baskets submerged in well‐aerated water with methylene blue (2.5 μg/ml) until hatching to prevent fungal growth. We used a full‐sib split‐brood design, where half of the embryos from each brood were reared at a low temperature (12°C) and the remaining embryos were reared at a high temperature (18°C).

F1 larvae were fed with newly hatched *Artemia nauplii*, microworms, and powdered food until juveniles were large enough to eat pelleted food (i.e. standard length of approximately 2 cm). They were then transferred to 10‐L tanks and kept at densities of 15–20 individuals. They were maintained at a constant water temperature of 12°C or 18°C (±0.5°C) from the embryonic stage to adulthood. As adults, they were fed a mixture of frozen bloodworms, *Mysis* shrimp and *Daphnia*. When these F1 sticklebacks were 10–12 months old, we used them to perform in vitro crosses as described above.

The resulting F2 offspring were reared at the same temperature as their F1 parents (Figure 3). They were also kept at a similar density and fed a similar diet to the F1 generation. We used F2 adults (aged 10–12 months) for the sociability trials described below. This breeding design, where fish were reared at a common temperature for two generations, allowed us to minimize parental and other epigenetic effects from their wild‐caught grandparents.

### 2.6 Experimental protocol for measuring sociability of F2‐generation sticklebacks

As before, we measured sociability by conducting binary choice tests in an experimental tank that was divided into three compartments with a camera mounted approximately 60 cm above the tank. In each sociability trial, the focal and stimulus fish were unfamiliar to each other but were always from the same thermal habitat of origin and reared at the same temperature (e.g., fish that were reared at 12°C and whose grandparents came from a cold habitat). Unlike our first experiment with wild‐caught fish, the F2‐generation fish were only tested at a single temperature (i.e., their rearing temperature), because our aim was to test the effects of rearing temperature and thermal habitat of origin (Figure 3). Thus, we tested each individual twice at their rearing temperature (12°C or 18°C) with 2–3 days between trials. Our sample sizes for this second experiment were between 34 and 38 individuals per thermal habitat per rearing temperature (total *n* = 145; Figure 3).

### 2.7 Video analysis

Videos from both experiments were analysed using the automated tracking software EthoVision XT (Noldus et al., 2001). The software logged the *x* and *y* coordinates of each focal fish for every frame of the video recording. We used this information to calculate sociability as the mean distance (cm) of the focal individual from the stimulus shoal over the 30 min trial (*sensu* Jolles et al., 2017; Killen et al., 2016, 2021).

### 2.8 Statistical analysis

To examine variation in sociability in wild‐caught sticklebacks, we ran separate linear mixed effects models for the allopatric and sympatric population pairs. We included thermal habitat of origin, acclimation temperature and their interaction as explanatory variables. The interaction term tested for the presence of a genotype‐by‐environment interaction (*G* × *E*), which would indicate environmentally induced phenotypic changes within individuals (i.e. phenotypic plasticity) that differ between genotypes. We also included fish identity as a random effect and the focal individual's standard length as an additional fixed effect to account for variation in body size within and between populations (Table S2). Similarly, to examine sociability in F2‐generation sticklebacks, we ran a linear mixed effects model with fish identity as a random effect and the following factors as fixed effects: focal fish size, thermal habitat of origin, rearing temperature, and the interaction between thermal habitat and rearing temperature. Lastly, we used the ‘rptR’ package (Stoffel et al., 2017) to estimate repeatability in sociability across the two trials for each wild‐caught and F2‐generation individual.

All statistical analyses were performed using R version 3.5.1 (R Core Team, 2018), and figures were generated using the ‘ggplot2’ package (Wickham, 2016). Mixed effects models were run using the ‘lme4’ package (Bates et al., 2015) and were fitted using maximum likelihood methods. *p*‐values were obtained by likelihood ratio tests of the full model with the explanatory variable in question against a second model without the variable in question. The level of statistical significance for all tests was *a* = .05.

## 3 RESULTS

### 3.1 Sociability in wild‐caught fish from warm and cold habitats

**FIGURE 4:**
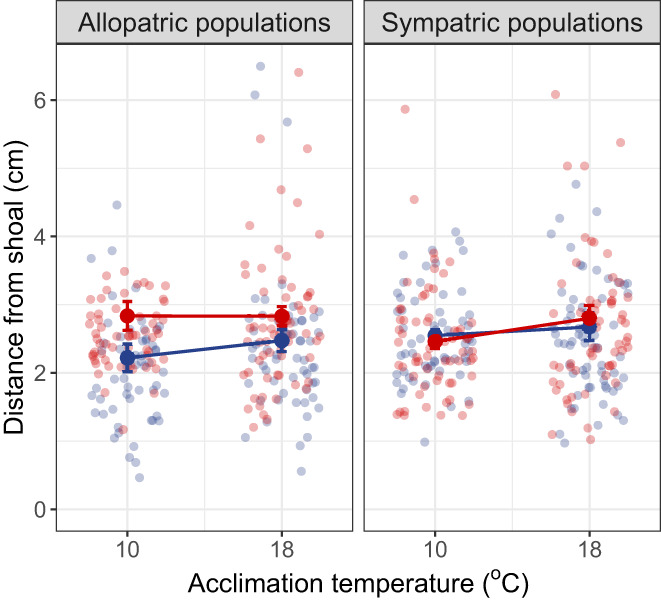
Mean (±SE) distance of a focal fish from the shoal of stimulus fish, as a measure of sociability for wild‐caught sticklebacks collected from cold (blue) and warm (red) habitats. Smaller values on the y‐axis indicate a greater degree of sociability. Each individual was tested twice at a low temperature (10°C) and twice at a high temperature (18°C) after acclimation of at least 1 month at the new temperature conditions. We found that fish from the warm habitat were less social than those from the cold habitat in the allopatric populations (*p* = .026) but not the sympatric populations (*p* > .99).

When comparing the allopatric populations, we found that sociability was lower in fish from the warm habitat than those from the cold habitat (Estimate = 0.40, SE = 0.18, *t* = 2.23, *p* = .026; Figure 4). However, fish did not adjust their social behaviour depending on the water temperature at which they were acclimated and tested (Estimate = 0.02, SE = 0.02, *t* = 0.81 *p* = .42; Figure 4). There was also no evidence for an interaction between thermal habitat of origin and acclimation temperature on sociability (*G* × *E*: Estimate = −0.02, SE = 0.04, *t* = −0.52, *p* = .60). Lastly, focal fish size did not have a significant effect on sociability (Estimate = −0.03, SE = 0.02, *t* = −1.31, *p* = .19). Fish from the allopatric populations showed repeatability in their sociability at a high temperature (*R* = 0.23, *p* = .008) but not at a low temperature (*R* = 0.013, *p* = .47).

In contrast to our results for the allopatric populations, there was no difference in sociability between fish from the warm versus cold habitat in the sympatric populations (Estimate = 0.002, SE = 0.22, *t* = 0.01, *p* > .99; Figure 4). Sociability was also not influenced by acclimation temperature (Estimate = 0.03, SE = 0.02, *t* = 1.60, *p* = .11), the interaction between thermal habitat of origin and acclimation temperature (*G* × *E*: Estimate = 0.03, SE = 0.03, *t* = 0.95, *p* = .35), or focal fish size (Estimate = −0.02, SE = 0.01, *t* = −1.69, *p* = .12). There was significant repeatability across trials when fish were acclimated to a low temperature (*R* = 0.28, *p* = .003) but not when acclimated to a high temperature (R = 0.072, *p* = .20).

### 3.2 Sociability in F2‐generation fish reared at low and high temperatures

**FIGURE 5:**
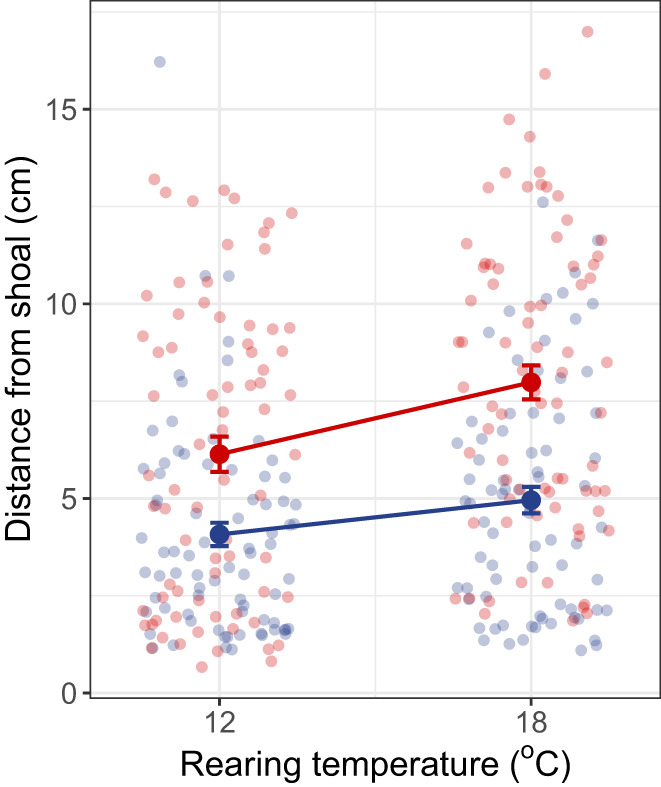
Mean (±SE) distance of a focal fish from the shoal of stimulus fish, as a measure of sociability for lab‐reared sticklebacks reared for two generations at a low or a high water temperature (12°C and 18°C, respectively). Smaller values on the *y*‐axis indicate a greater degree of sociability. For this common garden experiment, we used sticklebacks from the allopatric warm‐cold population pair. Fish whose grandparents originated from the cold habitat are indicated in blue, and those whose grandparents originated from the warm habitat are indicated in red. Solid lines between mean values represent population‐level reaction norms across rearing temperatures. We found that fish whose grandparents came from a warm habitat were less social than those whose grandparents came from a cold habitat (*p* < .0001). There was also an effect of rearing temperature on sociability: fish were less social when reared at a high temperature than when reared at a low temperature (*p* = .046).

In our second experiment, we used F2‐generation fish whose grandparents originated from the allopatric warm and cold habitats; these F2 fish were reared at a low or high temperature for two generations (Figure 3). We found that fish whose grandparents came from a warm habitat were less social than those whose grandparents came from a cold habitat regardless of the temperature at which the F2 fish were reared (Estimate = 2.69, SE = 0.44, *t* = 6.15, *p* < .0001; Figure 5). This result is consistent with the social behaviour we observed in the wild‐caught individuals, suggesting that the observed sociability differences in wild fish are heritable. However, there was also an effect of rearing temperature on sociability (Estimate = −0.92, SE = 0.47, *t* = −1.98, *p* = .046). Fish were less social when reared at a high temperature than when reared at a low temperature (Figure 5). This effect of rearing temperature was observed both in fish with grandparents from warm habitats and those with grandparents from cold habitats (thermal habitat × rearing temperature: Estimate = −0.86, SE = 0.87, *t* = −0.98, *p* = .32). There was also an effect of body size with larger fish being more sociable (Estimate = 1.10, SE = 0.45, *t* = 2.45, *p* = .014). Lastly, there was significant repeatability in the sociability behaviour of F2‐generation fish across the two trials, both when reared at a low temperature (*R* = 0.49, *p* < .0001) and when reared at a high temperature (*R* = 0.36, *p* < .001).

## 4 DISCUSSION

A recent review by Moss and While (2021) concluded that ‘we lack empirical evidence that explicitly tests the effects of temperature on social behaviour’. Here, we provide evidence for a causal effect of rearing temperature on social behaviour. Our first experiment showed that (i) in allopatric but not sympatric populations, wild‐caught fish from a warm habitat were less social than those from a cold habitat, (ii) there was no phenotypic plasticity in sociability in response to acclimation to two thermal environments, and (iii) the repeatability of sociability differed between fish acclimated to a low versus a high temperature, suggesting that temperature could affect the ability of selection to act on sociability. Using a common garden breeding design, our second experiment showed that sociability is likely to be heritable but also influenced by rearing temperature. When reared under a common temperature, fish whose grandparents came from a warm habitat were less social than those whose grandparents came from a cold habitat, indicating heritability of sociability. In addition, regardless of their origin, fish that were reared at a high temperature (18°C) were less social than those reared at a low temperature (12°C), suggesting that thermal conditions experienced in early life may play an important role in influencing social behaviour in adulthood.

The main finding of our first experiment was that wild‐caught fish from the allopatric warm habitat were less social than those from the allopatric cold habitat, but there was no such difference between thermal habitats in the sympatric population pair. Since we only found an effect in one population pair, a possible explanation for this pattern is that the observed differences in sociability in the allopatric populations are not a result of the thermal habitat itself. For example, they may be a result of genetic drift or some other component of the environment that differs between thermal habitats. An alternative explanation is that gene flow in the sympatric populations may constrain divergence in sociability or other correlated traits. Indeed, our preliminary population genomic analyses indicate extensive gene flow between thermal habitats in these sympatric populations (Costa et al., submitted), and a previous study on this study system showed little to no divergence in physiology between sympatric populations but significant divergence between allopatric populations (Pilakouta et al., 2020).

Under the latter scenario, the observed sociability differences between thermal habitats in the allopatric populations could be adaptive. We suggest three reasons reduced sociability may be advantageous in a warm environment. First, although sociality might allow individuals to find food patches more consistently (Ekman & Hake, 1988; Ruxton et al., 1995), there is stronger competition for discovered food items within groups (Webster & Hart, 2006). Thus, lower food availability or greater energetic demand in warmer waters may lead to increased food competition, making sociality more costly. Second, sticklebacks in warm habitats likely experience a lower risk of predation from freshwater piscivorous fish, which may be unable to cope with high temperatures (Eliason et al., 2011). Under these conditions, there is less benefit of being social in the form of improved predator detection and avoidance. Third, warmer environments are generally associated with a higher rate of disease transmission and increased virulence (Altizer et al., 2013; Harvell et al., 2002), making sociality more costly. Rising temperatures due to climate change could have similar effects on the balance between the costs and benefits of being social in natural populations; if higher temperatures increase the costs and reduce the benefits of sociality, we may thus expect animals to be less social in a warming world.

Our common garden experiment, which focused on the allopatric populations, found evidence for heritability in sociability. This suggests that it is possible for selection to act on sociability directly, but differences in sociability between thermal habitats could also be a by‐product of selection on a trait that is linked to both social behaviour and the thermal environment, such as metabolic rate. Our previous work on this study system has shown that wild sticklebacks from warm habitats tend to have a lower standard metabolic rate than those in cold habitats when measured at a common temperature (Pilakouta et al., 2020), and there is evidence for a link between standard metabolic rate and sociability in other species (Killen et al., 2016; but see Mathot et al., 2019). Nevertheless, in our first experiment, wild‐caught fish acclimated to a low vs a high temperature showed no evidence for plasticity in sociability in response to temperature, which seems to contradict this explanation. Acclimation to a higher temperature would have increased the metabolic rate of both warm‐ and cold‐habitat fish (Pilakouta et al., 2020), but this was not accompanied by a shift in sociability in either of the population pairs (Figure 4).

Our common garden experiment eliminated variation in many ecological factors, such as predation risk and food availability, that could drive the development of variation in sociability between temperature treatments. Yet, we still saw reduced sociability in fish reared at a higher temperature over two generations, regardless of their thermal habitat of origin (Figure 5). This suggests that the effects of temperature on sociability are at least partially mediated through intrinsic pathways, such as physiological or endocrine changes that occur throughout development (Moss & While, 2021), although it is worth noting that we also cannot eliminate the possible contribution of parental and other epigenetic effects from the F1 generation (Fuxjäger et al., 2019).

If the effects of temperature on social behaviour are mediated through bioenergetic or endocrine pathways, they may be relatively common in fishes and other ectotherms. In line with this, previous studies on various fish species have found that higher acclimation temperatures were associated with increased aggression and larger nearest‐neighbour distances in schools (Trinidadian guppy: Weetman et al., 1998, Weetman et al., 1999; walleye pollock: Hurst, 2007; Amazonian dwarf cichlid: Kochhann et al., 2015; brown trout: Colchen et al., 2017). Interestingly, many of the same endocrine signals that promote aggression have a reciprocal effect on sociability (Kelly & Vitousek, 2017). We encourage further work on this topic to determine whether the effects of temperature on social behaviour are widespread and how they vary among taxonomic groups (e.g., between ectotherms and endotherms). Understanding what drives variation in the extent of social behaviour is important given that social associations precede the emergence of complex social structures, such as group living and collective behaviour, with fitness consequences for individuals and populations (Maldonado‐Chaparro & Chaverri, 2021; van Schaik et al., 2003).

## 5 CONCLUSION

Sociability can influence an individual's vulnerability to predation, ability to find food, and disease risk, so it has important effects on individual fitness and group dynamics. To fully understand the evolution of social behaviour, we need to disentangle the influence of environmental factors (phenotypic plasticity), genetic factors (evolutionary responses), and their interaction. Our study found no evidence for plastic responses in the sociability of wild fish in response to temperature changes in adulthood. Instead, we show that (i) individuals originating from an allopatric warm habitat were less social than those from the adjacent cold habitat and (ii) sociability can be both heritable and influenced by rearing temperature. By demonstrating a causal effect of rearing temperature on sociability, this work provides novel insights into how temperature changes could influence social behaviour in a warming world.

## AUTHOR CONTRIBUTIONS

Natalie Pilakouta, Shaun S. Killen, Neil B. Metcalfe, Jan Lindström, and Kevin J. Parsons conceived and designed the study. Bjarni K. Kristjánsson and Skúli Skúlason helped coordinate fieldwork and provided supplies. Natalie Pilakouta and Kevin J. Parsons conducted the fieldwork. Shaun S. Killen provided the laboratory equipment. Joseph L. Humble helped with animal husbandry and provided logistical support. Natalie Pilakouta, Patrick J. O'Donnell, Amélie Crespel, and Marion Claireaux performed the experiment and ran the behavioural trials. Natalie Pilakouta, Patrick J. O'Donnell, Amélie Crespel, and Marie Levet did the video analysis. Natalie Pilakouta did the statistical analysis and wrote the manuscript. All authors edited the manuscript and gave final approval for publication.

## FUNDING INFORMATION

The study was funded by a Natural Environment Research Council Grant (NE/N016734/1) awarded to KJP, NBM, SSK, and JL, as well as a grant from the Wellcome Trust Institutional Strategic Support Fund (ISSF) awarded to NP. SSK was supported by a NERC Advanced Fellowship (NE/J019100/1) and a European Research Council Starting Grant (640004).

## DATA AVAILAIBILITY STATEMENT

The relevant data are available on the Dryad Digital Repository Repository (Pilakouta et al., 2022): https://doi.org/10.5061/dryad.1g1jwsv0v

## Supporting information


Table S1.
Table S2.
