# Supplementary material for: A warmer environment can reduce sociability in an ectotherm
Source: Glob Chang Biol. 2022 Oct 19;29(1):206–14. doi: 10.1111/gcb.16451 (PMC10092372; doi:10.1111/gcb.16451)
Supplement: Supplementary file 1 — Table S1. Table S2. [file GCB-29-206-s001.pdf]

Supplementary Table 1. Sampling locations and sample sizes of warm- and cold-habitat sticklebacks collected in March 2017. Distance refers to how far apart the warm-habitat and cold-habitat sample sites are for each population pair. The summer and winter temperatures listed are the average water temperatures recorded at each sampling location during the corresponding seasons. Sample sizes indicate the number of wild-caught fish from each sampling location that were used in our first experiment after being acclimated to 10ºC or 18ºC for at least one month.

**Population pair**	**Distance (m)**	**Water body**	**Thermal habitat**	**Winter temperature (ºC)**	**Summer temperature (ºC)**	**Sample sizes for wild-caught fish**	
						**Acclimated to 10ºC**	**Acclimated to 18ºC**
Allopatric	25	Unnamed (65°43'56"N 19°37'07"W)	Warm	10.1	22.4	31	33
			Cold	2.3	14.0	28	30
Sympatric	30	Áshildarholtsvatn (65°43'31"N 19°36'03"W)	Warm	12.5	24.1	30	33
			Cold	3.4	12.2	33	32

Supplementary Table 2. Mean (±SD) standard length (cm) of wild-caught and F2-generation sticklebacks used in this study.

Population pair	Thermal habitat	Wild-caught fish	F2 generation fish	
			Reared at 12°C	Reared at 18°C
Allopatric	Warm	4.73 ± 0.38	4.14 ± 0.61	4.79 ± 0.60
	Cold	5.00 ± 0.61	4.44 ± 0.68	4.86 ± 0.63
Sympatric	Warm	4.62 ± 0.60	—	—
	Cold	4.51 ± 0.61	—	—
